# Epidemic HI2 Plasmids Mobilising the Carbapenemase Gene *bla*_IMP-4_ in Australian Clinical Samples Identified in Multiple Sublineages of *Escherichia coli* ST216 Colonising Silver Gulls

**DOI:** 10.3390/microorganisms9030567

**Published:** 2021-03-10

**Authors:** Hassan Tarabai, Ethan R. Wyrsch, Ibrahim Bitar, Monika Dolejska, Steven P. Djordjevic

**Affiliations:** 1 Department of Biology and Wildlife Diseases, Faculty of Veterinary Hygiene and Ecology, University of Veterinary and Pharmaceutical Sciences Brno, Brno 612 42, Czech Republic; hassantarabai@gmail.com; 2 Central European Institute of Technology (CEITEC), University of Veterinary and Pharmaceutical Sciences Brno, Brno 612 42, Czech Republic; ibrahimbitar5@gmail.com; 3 iThree Institute, University of Technology Sydney, Sydney, NSW 2007, Australia; ethan.wyrsch@uts.edu.au; 4 Biomedical Center, Faculty of medicine in Pilsen, Charles University, Pilsen 323 00, Czech Republic; 5 Department of Clinical Microbiology and Immunology, Institute of Laboratory Medicine, The University Hospital Brno, Brno 625 00, Czech Republic

**Keywords:** anthropogenic pollution, ST216, Australian silver gull, urban birds, wildlife, whole genome sequencing, *Chroicocephalus novaehollandiae*

## Abstract

*Escherichia coli* ST216, including those that carry *bla*_KPC-2_, *bla*_FOX-5_, *bla*_CTX-M-15_ and *mcr-1*, have been linked to wild and urban-adapted birds and the colonisation of hospital environments causing recalcitrant, carbapenem-resistant human infections. Here we sequenced 22 multiple-drug resistant ST216 isolates from Australian silver gull chicks sampled from Five Islands, of which 21 carried nine or more antibiotic resistance genes including *bla*_IMP-4_ (*n* = 21), *bla*_TEM-1b_ (*n* = 21), *aac*(3)-*IId* (*n* = 20), *mph*(A) (*n* = 20), *catB3* (*n* = 20), *sul1* (*n* = 20), *aph*(3”)-*Ib* (*n* = 18) and *aph*(6)-*Id* (*n* = 18) on FIB(K) (*n* = 20), HI2-ST1 (*n* = 11) and HI2-ST3 (*n* = 10) plasmids. We show that (i) all HI2 plasmids harbour *bla*_IMP-4_ in resistance regions containing In809 flanked by IS*26* (HI2-ST1) or IS*15DI* (HI2-ST3) and diverse metal resistance genes; (ii) HI2-ST1 plasmids are highly related to plasmids reported in diverse Enterobacteriaceae sourced from humans, companion animals and wildlife; (iii) HI2 were a feature of the Australian gull isolates and were not observed in international ST216 isolates. Phylogenetic analyses identified close relationships between ST216 from Australian gull and clinical isolates from overseas. *E. coli* ST216 from Australian gulls harbour HI2 plasmids encoding resistance to clinically important antibiotics and metals. Our studies underscore the importance of adopting a one health approach to AMR and pathogen surveillance.

## 1. Introduction

Bacterial isolates resistant to antimicrobials have been isolated from wildlife [1] and there are concerns that wild animals act as critically important vectors and reservoirs for antimicrobial resistant bacteria and antibiotic resistance genes (ARGs) [2,3]. Migratory birds have the potential to spread multidrug resistant (MDR) bacteria and ARGs [4,5] posing a significant threat to biosecurity, particularly in countries that practice sound antimicrobial stewardship [1,6]. An increasing number of migratory birds have been found to host antimicrobial resistant bacteria with resistance to diverse antibiotics including those referred to as critically important (CIA) to human health such as the extended spectrum β-lactams, fluoroquinolones, carbapenems [7,8,9] and colistin [10,11,12,13].

Plasmids are vehicles that capture, assemble, maintain and spread ARGs genes, heavy metal resistance genes and virulence-associated genes (VAGs) [6] and provide flexibility to bacterial genomes by the diverse genetic cargo they carry [14]. Genes encoding resistance to antibiotics, heavy metals and virulence genes often coassemble on the same plasmid, mediated in part by the activity of insertion elements such as IS*26* [15,16,17,18]. These factors allow the emergence of lineages that carry complex resistance regions and virulence gene profiles [14,19]. As such there is an urgent need to address the shortage of completed plasmid sequences in public databases and identify plasmids that carry virulence and antibiotic resistance genes [15,17,20] and plasmid hybrids [16,21]. Hybrid *Escherichia coli* carrying combinations of virulence genes from different pathovars are increasingly recognised as an emerging threat to human and animal health [22].

Dissemination of emergent and dominant multidrug resistant bacterial clades is a major driving force behind the global spread of antibiotic-resistant bacteria [14,23]. *E. coli* ST216 is known to carry genes encoding resistance to a broad range of antibiotics including those of clinical relevance [24,25,26]. However, little is known about ST216 virulence and antibiotic resistance gene cargo that it carries and the hosts it occupies. *E. coli* ST216 belongs to ‘commensal’ phylogroup A. With the exception of ST10 and STs belonging to clonal complex 10, phylogroup A has not been widely recognised as pathogenic in humans or non-human animals [27]. However *E. coli* belonging to the commensal phylogroups A and B1 are able to acquire virulence genes [17] and ARGs [28,29] and cause disease [17]. While ST216 is not one of the 20 most frequently reported *E. coli* STs on a global scale [27], MDR *E. coli* isolates of ST216 are recovered from humans with clinical infections [25,26,30,31]. Notably, *Klebsiella pneumonia* carabapenemase (KPC)-producing *E. coli* ST216 was linked with a large (125 isolates) and recalcitrant outbreak in Central Manchester University Hospital in the United Kingdom in 2015 [24]. During that episode, WGS showed that IncHI2 plasmids carrying *bla*_KPC_ spread from ST216 to other *E. coli* STs as well as other Enterobacteriaceae. A notable feature of the UK outbreak was the recalcitrant nature of the contamination and the extraordinary measures taken to eliminate *bla*_KPC_^+^ ST216 from several cardiac wards by replacing plumbing infrastructure, a measure that only partially alleviated subsequent episodes of infection. The *bla*_KPC-2_ gene in the UK outbreak was a component of a Tn*4401a* transposon [24], known for its enhanced KPC expression [32] and this study, while notable, represents the only one retrieved by a PubMed search using “*E. coli* ST216” as a search parameter at the time of writing (26/12/2020). However, *bla*_KPC-2_ was shown to be associated with a Tn*4401g* transposon located on an N plasmid in a clinical isolate of *E. coli* ST216 in Israel [25] and hospital-acquired *E. coli* ST216 carrying *bla*_FOX-5_ (serine β-lactamase with a substrate specificity for cephalosporins) linked to an IncC plasmid were recovered from a senior patient at an intensive care unit in the University of Maryland Medical Center in the United States [26]. In a possible episode of patient to family transmission, two *bla*_CTX-M-15_-positive *E. coli* ST216 isolates were identified in a family member of a patient at Tel-Aviv Sourasky Medical Center in Israel [30] and colistin resistance was reported in an *E. coli* ST216 harbouring *mcr-1* in a urine sample from a patient in Italy [31]. In the environment, *E. coli* ST216 carrying *bla*_KPC-2_ have been found on an R plasmid from a river ecosystem in Barcelona, Spain [33]. These episodes point to ST216 *E. coli* being proficient in the capture and dissemination of genes encoding β–lactamases and carbapenemases and suggest aquatic environments and both domestic and industrial wash basins and sinks may be an ideal niche. While speculative, the ability of ST216 to form recalcitrant biofilms in hospital wastewater pipes [24] is consistent with this view.

The silver gull typically breeds in large colonies on offshore islands and frequents urban environments including garbage dumps, shopping centres, railway stations, municipal parks and promenades along large river embankments. The Australian silver gull is known to harbour multiple drug-resistant *E. coli* [34,35] and *Salmonella enterica* spp. [36] highlighting hotspots where drug-resistant bacteria accumulate in urban environments [37,38]. In this study, we conducted whole genome sequencing of 22 MDR *E. coli* ST216 isolates from silver gulls (*Chroicocephalus novaehollandiae*) nesting on Big Island, 60 km south from Sydney in Australia, one of Australia’s largest silver gull breeding sites. We investigated their phylogeny and serotype composition and determined the antibiotic and virulence gene cargo they carry. Long read sequencing of plasmid DNA enabled the determination of the complete sequence of several plasmids that carry genes encoding resistance to CIA. Phylogenetic analysis of 123 MDR *E. coli* ST216 isolates and their plasmid content enabled an assessment of the spread of these isolates and their associated mobile elements and ARGs both within Australia and globally, and shed light on the potential risks they present to human and animal health.

## 2. Methods

### 2.1. E. coli ST216 Collection from Gulls

Bacterial isolates were obtained from our previous study investigating silver gulls (*Chroicocephalus novaehollandiae*) as carriers of antibiotic resistant bacteria in New South Wales, Australia [34]. In that study, cloacal samples (*n* = 504) from gull chicks were collected at three locations (Five Islands, White Bay in Sydney and Montague Island) and 27 IMP-producing *E. coli* ST216 were obtained, all originating from silver gulls at Five Islands. Based on PFGE genomic and plasmid profiles, six representative isolates were selected for further analysis. An additional 16 *E. coli* ST216 isolates were obtained by cultivation of primary cloacal samples enriched overnight in buffered peptone on MacConkey agar with cefotaxim (2 mg/L) or ciprofloxacin (0.05 mg/L). Their sequence types were determined following WGS of all *E. coli* isolates (*n* = 448) obtained from silver gulls at the three sampling locations (our unpublished data). A total of 22 *E. coli* ST216 isolates, all originating from gulls in Five Islands, were obtained and subjected to WGS (Supplementary Table S1).

#### Whole Genome Sequencing

Genomic DNA of 22 gull ST216 was isolated using NucleoSpin^®^ Tissue kit (Macherey-Nagel GmbH & Co, Duren, Germany). DNA libraries were prepared using Nextera XT DNA library preparation kit and sequenced on a NovaSeq (Illumina, San Diego, CA, USA) platform. Assembly of obtained short reads was performed using Shovill v0.9.0 software [39].

Genomic DNA from *E. coli* CE1537 was selected for long read sequencing to obtain a complete *E. coli* ST216 reference sequence. Whole-genome DNA was extracted using NucleoSpin^®^ Tissue kit (Macherey-Nagel GmbH & Co, Duren, Germany) and library preparation was performed using microbial multiplexing based on the manufacturer’s recommendation. The DNA was sheared using g-tubes (Covaris, Woburn, MA, USA) but size selection was not performed for library preparation. The Sequel 1 platform (Pacific Biosciences, Menlo Park, CA, USA) was used for long-read sequencing. Sequence assembly was carried with HGAP v4.0 software [40] and resulted in 11 contigs with an average 203-fold coverage. The incomplete sequence of an HI2-ST1 plasmid was identified on a separate contig, therefore, long-read sequencing using plasmid DNA of CE1537 was performed as described below.

Long-read sequencing of plasmid DNA (pDNA) extracted from isolates CE1537 and CE1681 was carried out to generate complete plasmid sequences. Plasmid DNA was extracted using a QIAGEN^®^ midi kit (Qiagen, Hilden, Germany) and library preparation using a microbial multiplexing protocol was performed as described above. The Sequel 1 platform (Pacific Bioscinces, Menlo Park, CA, USA) was used for pDNA sequencing followed by assembly of obtained reads with SMRT LNK v8.0 software (Pacific Biosciences, Menlo Park, CA, USA). This resulted in the assembly of six circular contigs from isolate CE1537 (pCE1537-A to pCE1537-F) with an average 317-fold coverage and six contigs from isolate CE1681 (pCE1681-A to pCE1681-F) with an average 326-fold coverage (Supplementary Table S2).

FastQC (https://www.bioinformatics.babraham.ac.uk/projects/fastqc/) was used for quality control of obtained short reads and long read sequences (accessed on 15 February 2021-Table 1).

### 2.2. E. coli ST216 Metadata

All *E. coli* ST216 short reads were deposited on EnteroBase in the *Escherichia*/*Shigella* database and on Genbank (see Table 1 for barcode numbers and accession numbers, respectively) [41]. Short read sequences of ST216 isolate CE1537 were discarded due to contamination.

Long read sequence of isolate CE1537 was deposited on GenBank (accession number (AN): JABBCF000000001). Similarly complete and closed plasmids pCE1537-A (AN: MT232840), pCE1537-B (AN: MT162140), pCE1537-C (AN: MT162141), pCE1537-D (AN: MT162142), pCE1537-E (AN: MT162143), pCE1537-F (AN: MT162144), pCE1681-A (AN: MT180430), pCE1681-B (AN: MT180431), pCE1681-C (AN: MT180432), pCE1681-D (AN: MT180433), pCE1681-E (AN: MT180434) and pCE1681-F (AN: MT180435) were deposited in GenBank. Quality control on long read sequences of genomic DNA 

**Figure 1:**
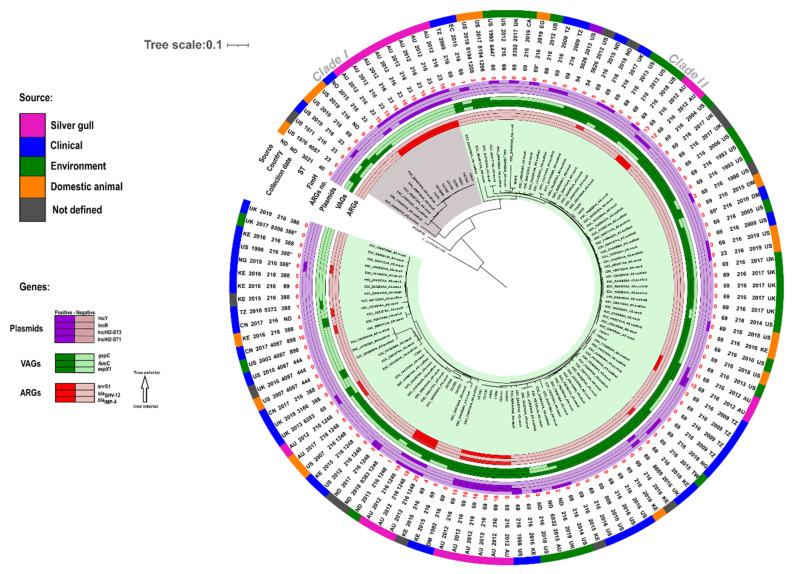
Clonal relationship of *Escherichia coli* ST216 isolates from silver gulls (*n* = 22) at Five Islands and international related isolates (*n* = 100).

For phylogenetic analysis genomes of *E. coli* ST216 (*n* = 99) from the EnteroBase *Escherichia*/*Shigella* database with one allele mismatch were selected and their assembly barcode used in the constructed phylogenetic tree (Figure 1). *E. coli* ST216 sequences with no sample collection date were excluded. In addition, one *E. coli* ST216 from Genbank with assembly and accession numbers GCA_002263825.1 and NNAL00000000 respectively, was used in ST216 phylogenetic analysis. 

#### 2.2.1. WGS Analysis

Publicly available tools were used to analyse short and long read sequences of *E. coli* ST216 gull and Enterobase sequences. The affiliation to sequence type, serotype and *fimH* type were confirmed using MLST (v2.0), SerotypeFinder (v.2.0) and FimTyper (v.1.0), respectively (accessed on 13 August 2020 and available at https://cge.cbs.dtu.dk/services/). *E. coli* ST216 phylogroup was assigned using Clermont Typing (accessed on 13 August 2020 and available at http://clermontyping.iame-research.center/). ResFinder v.3.2 (accessed on 15 August 2020 and available at https://cge.cbs.dtu.dk/services/) and CARD [42] and Virulence Factor DataBase (VFDB) [43] were used to identify antibiotic resistance and virulence genes, respectively. Plasmid replicons and plasmid ST were determined using PlasmidFinder (v.2.0) and pMLST (v.2.0) respectively (accessed on 15 August 2020 and available at https://cge.cbs.dtu.dk/services/). Automated annotation of all sequences was generated by RASTtk [44].

Complete sequences of pDNA from isolates CE1537 and CE1681 (Supplementary Table S2) were assessed for plasmid types, ARGs, VAGs and phages. The metal resistance genes content of closed plasmids was evaluated using BacMet (accessed on 25 August 2020 and available at http://bacmet.biomedicine.gu.se/blast/blast_link.cgi). Automated annotation of complete plasmid sequences was generated by RAST-tk [44] followed by manual curation using SnapGene^®^ v5.0.6 (GSL Biotech LLC, Chicago, IL, USA) and a BLASTn online tool (NCBI, Rockville, MD, USA). Insertion sequences and phage-associated genes within closed contigs (Supplementary Table S2) were searched by ISFinder [45] and PHASTER [46].

#### 2.2.2. Antibiotic Susceptibility Testing of Gull Isolates

Susceptibility to 15 different antibiotics of gull-sourced *E. coli* ST216 isolates was tested using the disk diffusion method according to European Committee on Antimicrobial Susceptibility Testing (EUCAST) recommendations [47] using the following antibiotic discs (Oxoid, Hants, UK): amoxicillin-clavulanic acid (20–10 µg), ampicillin (10 µg), cefalotin (30 µg), ceftazidime (30 µg), chloramphenicol (30 µg), ciprofloxacin (5 µg), ertapenem (10 µg), gentamicin (10 µg), imipenem (10 µg), nalidixic acid (30 µg), meropenem (10 µg), sulphonamide compounds (300 µg), streptomycin (10 µg), tetracycline (30 µg) and trimethoprim-sulfamethoxazole (1.25/23.75 µg). Measuring and interpretation of inhibition zone diameters of the tested isolates was performed according to EUCAST breakpoints [48] or using breakpoints defined by CLSI 2017 [49] for antibiotics (azithromycin, cefazolin, tetracycline, nalidixic acid, sulphonamide compounds and streptomycin) with no defined breakpoints in EUCAST 2019 [48]. *E. coli* ST216 isolates were tested for susceptibility to colistin using colispot test [50]. AmpC, extended-spectrum beta-lactamase and carbapenemase production in *E. coli* ST216 isolates was assessed using AmpC, ESBL and Carbapenemase Set D72C (Mast Diagnostics, Merseyside, UK) and carbapenemase production was confirmed with matrix-assisted laser desorption ionisation-time of flight mass spectrometry [51]. ST216 that are nonsusceptible to at least one antibiotic in three or more antibiotic classes were deemed to be multidrug-resistant [52]. 

#### 2.2.3. Phylogenetic Analysis

Single nucleotide polymorphisms (SNPs) (Figure 1 and Supplementary Figure S1) were identified and used for phylogenetic analysis of ST216 using CSI Phylogeny 1.4 [53]. A second SNP analysis (Supplementary Figure S2) including only gull *E. coli* ST216 isolates sequenced in this study was also performed. Phylogenetic trees were visualised with iTOL v4 [54]. Comparison and alignment of long- and short-read sequences with *E. coli* reference genomes from K12-MG1655 and *E. coli* ATCC8739 (GenBank Accession no.: U00096.3 and NZ_CP022959.1, respectively) were performed using progressiveMauve [55]. 

#### 2.2.4. Phylogenetic Analysis of Complete and Closed Plasmids

To investigate the distribution of closed contigs (pCE1537-A, pCE1537-B, pCE1681-A, pCE1681-B, pCE1681-D) within the genome sequences of *E. coli* ST216 sourced from gulls, heat maps were generated and visualised using the chooklord pipeline (accessed on 4 March 2021 and available at https://github.com/maxlcummins/chooklord [56]) with closed plasmids as reference (Figure 5A,B and Supplementary Figure S3A,E). A BLASTn search was performed and complete plasmid sequences with an identity threshold of ≥99% and a query coverage threshold of ≥91% were selected for further analysis (Table 2). A different coverage threshold (86%) was set for investigating plasmid pCE1681-E because pEc1677, which showed the highest detected coverage (86%) with pCE1681-E, was selected from a BLASTn analysis. BRIG v0.95 software [57] and SnapGene^®^ v5.0.6 software (GSL Biotech LLC, Chicago, IL, USA) were used to perform comparisons of plasmid sequences. IS*26-*associated regions of plasmids pCE1537-A were subjected to further analysis using Easyfig v2.2.3 [58] for comparison and visualisation.

#### 2.2.5. Transferability of HI2 Plasmids

The MDR profile of sequenced *E. coli* ST216 isolates (CE1537 and CE1681) prevented the performance of a direct conjugation assay with a suitable recipient (employing a unique selection marker from the donor cells). We performed a multiphase test to circumvent this obstacle and check the conjugative properties of the resolved HI2 plasmids. HI2 plasmids were selected for transferability testing due to their carriage of *bla*_IMP-4_ which was a primary focus for this study. Plasmid DNA was extracted from donor cells using the QIAGEN^®^ midi kit (Qiagen, Hilden, Germany) and then transferred via electroporation to plasmid-free *E. coli* Top10 cells. Transformants positive for HI2 plasmid were selected on LB agar supplemented with cefotaxime (2 mg/L) and incubated overnight at 37 °C. The presence of *bla*_IMP_ and the HI2 plasmid in transformants was confirmed by PCR [34] and replicon typing [59], respectively. Total cellular DNA from transformants was digested with S1 nuclease and subjected to pulsed-field gel electrophoresis to confirm plasmid carriage. Conjugative transfer of HI2 positive transformants to a plasmid-free, rifampicin and sodium-azide resistant *E. coli* MT102 recipient strain [60] was performed using filter-mating method with incubation for 4 h at 28, 30 and 37 °C followed by selection of transconjugants on LB agar plates supplied with cefotaxime (2 mg/L), rifampicin (25 mg/L) and sodium azide (100 mg/L) and incubation overnight at 37 °C. The presence of *bla*_IMP_ and the HI2 plasmid in four transconjugants was confirmed by PCR [34] and replicon typing [59], respectively.

## 3. Results

### 3.1. Population Structure of Gull E. coli ST216

*E. coli* ST216 represents 8.4% of all *E. coli* isolates (*n* = 262) obtained from cloacal samples at Big Island and 5% of the 448 *E. coli* recovered from the three sampling sites (Five Islands, Sydney and Montague Island) (Supplementary Table S1). All 22 *E. coli* ST216 isolates that were sequenced are phylogroup A. In silico O:H typing identified two serotypes: O45:H4 (*n* = 1) and O154:H4 (36%, *n* = 8). Thirteen isolates were O-non-typable with flagella type H4 (9%, *n* = 2), O-non-typable with H-non-typable (36%, *n* = 8) and O45 with H-non-typable (14%, *n* = 3). *fimH* profiling revealed three *fimH* types including *fimH*23 (36%, *n* = 8), *fimH*69 (45.5%, *n* = 10), and *fimH*1248 (18%, *n* = 4) (Supplementary Table S1).

### 3.2. Phylogenetic Analysis of E. coli ST216

A SNP-based phylogenetic tree was constructed with genomes of 122 ST216 including the 22 *E. coli* ST216 isolates from silver gulls and 20 isolates with STs that carried a variant in a single multilocus sequence allele from ST216 (Figure 1). All isolates, including those from silver gulls, segregated into two main clades with significant diversity between them (3000–9000 SNP variants). Silver gull isolates also divided into two main SNP cluster groups (SCG) and six phylogenetic subgroups interspersed throughout the SNP-tree (Figure 1). Isolates from silver gulls (*n* = 22) were phylogenetically diverse showing a minimum of six and maximum of 8505 SNPs differences. The first SNP cluster (SCG1) which included eight *E. coli* ST216 isolates that differed only by 11–52 SNPs was located on a distinct branch (clade I in Figure 1) that included a handful of clinical isolates from the USA and a single clinical isolate from Norway. The gull cluster in clade I was most closely aligned (maximum of 283 SNPs difference between SCG1 and ESC_QA4689AA_AS) to the clinical isolate from Norway (ESC_QA4689AA_AS) (Figure 1). ST216 gull isolates in the second cluster (SCG2, *n* = 14) were more distinct (6–2347 SNPs difference) and included isolates from the clinic, environment, and domestic animals (clade II in Figure 1). Gull isolate 1720H in clade II was related (163–259 SNPs difference) to domestic animal isolates (ESC_TA2295AA_AS and ESC_GA6917_AS) from Australia and the USA, clinical isolates (ESC_ZA4597AA-AS and ESC_AA8402AA_AS) from Kenya and the USA and to an environmental isolate (ESC_GB5355AA_AS) of unknown origin.

*E. coli* ST216 isolates from silver gulls had a higher content of plasmids and ARGs (mean of 4.5 plasmids and 15 ARGs in silver gulls) compared to international ST216 isolates (mean of 1.7 plasmids and 1 ARG; Supplementary Figure S1), an observation that is consistent with selection on media containing antibiotics. In contrast to SCG1 and SCG2, HI2 plasmids were absent from almost all of the 100 international isolates. FIB(K) plasmids were the most common plasmids in international isolates (31%, *n* = 31) and were dominant in SCG1 and SCG2 (91%, *n* = 20). R plasmids were present in international isolates (11%, *n* = 11) and SCG2 (50%, *n* = 7) but absent from SCG1. Similarly, X5 plasmids were dominant in SCG2 (71%, *n* = 10) and not detected in SCG1. Resistance genes *bla*_IMP-4_, *bla*_SHV-12_ and *dfrA19* were only identified in SCG1 and SCG2 and were not detected in any international isolate (Supplementary Figure S1) indicating that HI2 plasmids harbour genes encoding these CIA. Other ARGs including *qnrS1*, *aac(6’)-Ib-cr* and *dfrA14* were identified both in SCG2 (71%, 93%, 64%, *n* = 10, *n* = 13, *n* = 9, respectively) and in international isolates (6%, 10% and 4%; *n* = 6, *n* = 10, *n* = 4, respectively) (Supplementary Figure S1). VAGs in SCG1, SCG2 and international isolates were common to *E. coli* species (Supplementary Figure S1). However, several VAGS were only present in SCG2 and international isolates including putative type III secreted effector *espX1*, fimbrial associated genes *fimC*, *fimD, fimE* and f*imI* and general secretion pathway genes *gspC*, *gspD*, *gspE*, *gspF*, *gspI* and *gspK* (Supplementary Figure S1).

### 3.3. Virulence Associated Genes (VAGs) of Gull Isolates

**Figure 2:**
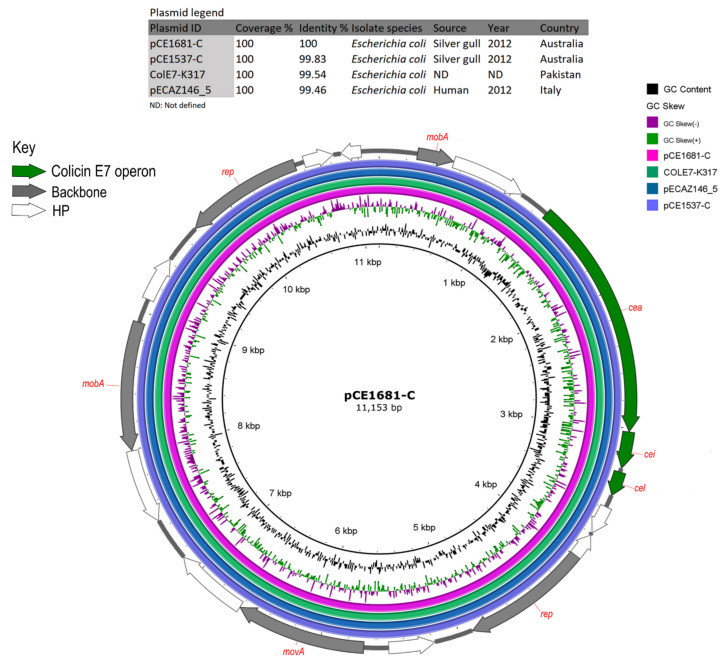
BRIG comparison of colicin E7 positive Col156 plasmid pCE681-C with similar plasmid sequences retrieved from GenBank. In the key, HP: hypothetical protein.

Carriage of virulence genes by the 22 ST216 isolates was unremarkable. Between 18 and 33 VAGs previously described among *E. coli* were identified in ST216 sequences. VAGs included enterobactins and elements of type II/III secretory systems, type I fimbriae regulators, ferric enterobactin transport system and general secretion pathway proteins (Supplementary Table S1). In isolate CE1537, a flagellin gene *fliC* was located on an FIA(HI1) plasmid (pCE1537-B) with a *fliC* repressor gene residing between two IS elements (Supplementary Figure S4-B and Supplementary Table S2). In isolate CE1681, a colicin E7 operon that includes the colicin E7 protein, colicin E7 immunity protein and a colicin E7 lysis protein is located on a Col156 plasmid (pCE1681-C) (Figure 2). In the same isolate (CE1681), a haemolysis expression-modulating protein (Hha) is located on X5 plasmid pCE1681-E (Figure S4-C).

### 3.4. Antibiotic Resistance Phenotypes and Genes of Gull Isolates

The collection of 22 sequenced isolates present variable MDR phenotypes with resistance ranging from 4 to 15 antibiotics (Supplementary Table S1). Most isolates (95%, 21/22) are carbapenemase producers (Supplementary Table S1). All isolates are resistant to streptomycin and sulphonamides (100%, 22/22) with 95% (21/22) of the isolates resistant to ampicillin, trimethoprim/sulfamethoxazole, cefalotin, ceftazidime and amoxicillin/clavulanic acid. Resistance to chloramphenicol and gentamicin were both observed in 91% (20/22) of the isolates and most ST216 isolates (19/22, 86%) are also resistant to tetracycline. Additional resistance to nalidixic acid, ertapenem, imipenem and meropenem was detected in 68% of isolates (15/22) while resistance to ciprofloxacin was present in 45% (10/22) of isolates. In summary, almost all ST216 are classified as multidrug resistant and express resistance to clinically important antibiotics.

We identified a total of 30 ARGs in the sequenced population of gull *E. coli* ST216 isolates and the strains carry between 7 and 20 ARGs each (Supplementary Table S1). A total of 21 out of 22 (95.45%) isolates carried nine or more ARGs. The most common ARGs among the ST216 isolates included *bla*_IMP-4_ (95.5%, *n* = 21), *bla*_TEM-1b_ (95.5%, *n* = 21), *aac*(3)-*IId* (91%, *n* = 20), *mph*(A) (91%, *n* = 20), *catB3* (91%, *n* = 20), *sul1* (91%, *n* = 20), *aph*(3”)-*Ib* (82%, *n* = 18) and *aph*(6)-*Id* (82%, *n* = 18) (Supplementary Table S1). The quinolone resistance genes *qnrA1* (36%, *n* = 8), *qnrS1* (45.5%, *n* = 10) and *aac*(6*’*)-*Ib-cr* (45.5%, *n* = 10) were less frequent. All ST216 isolates carried a class 1 integrase *intI1* and IS*26* (Supplementary Table S1). The resistance phenotype of most ST216 isolates correlated with their genotype except for isolate CE1681 and 1720H which exhibited phenotypic resistance to trimethoprim and chloramphenicol, respectively, with the absence of corresponding ARGs (Supplementary Table S1).

Most *E. coli* ST216 (95.5%) have at least one plasmid-mediated quinolone resistance gene (21/22) with only nine isolates expressing phenotypic resistance to ciprofloxacin and nalidixic acid. Of the 22 ST216 isolates, six are resistant only to nalidixic acid, one isolate is resistant only to ciprofloxacin while six isolates are susceptible to ciprofloxacin and nalidixic acid (Supplementary Table S1). Resistance to ertapenem, meropenem and imipenem was detected in 15/21 *bla*_IMP-4_-positive *E. coli* ST216 isolates (Supplementary Table S1). Known chromosomal *gyrA* mutation S83L that confers resistance to nalidixic acid and ciprofloxacin was identified in isolates 1556m1, 1548R1 and 1605m2 (Supplementary Table S1) [61]. Moreover, several chromosomal mutations with unknown effect were detected in *16S_rrsB*, *16S_rrsC*, *16S_rrsH*, *23S, pmrB, parC* and *gyrA* genes for isolates CE1537 and CE1586. Isolate CE1586 had an additional chromosomal mutation in *pmrA* gene. 

**Figure 3:**
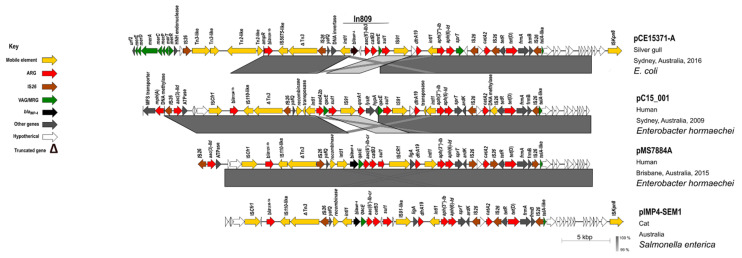
Comparison of IS*26*-composite transposon region and its background within IncHI2-ST1 plasmids from wildlife (pCE1537-A), companion animal (pIMP4-SEM1) and humans (pMS7884a and pC15_001). In the key, ARG: antibiotic-resistance gene and VAG/MRG: virulence associated gene/metal resistance gene.

**Figure 4:**
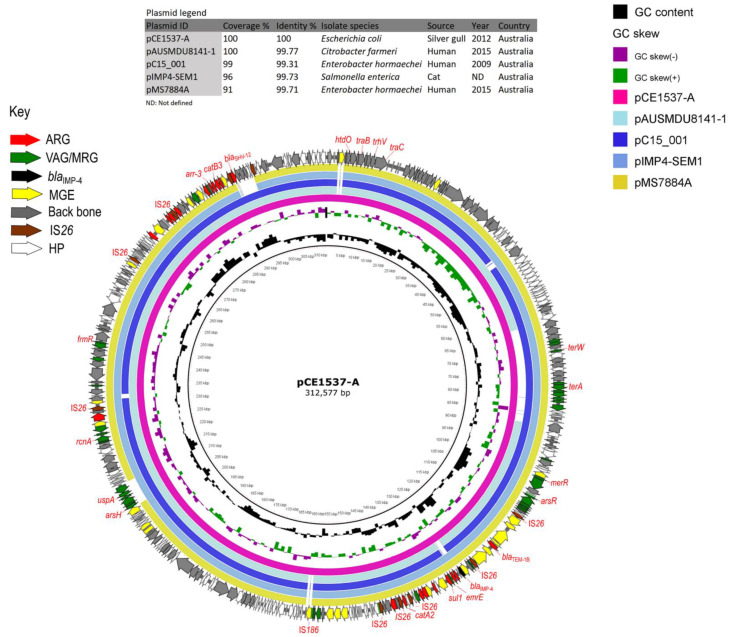
BRIG comparison of *bla*_IMP-4_-positive IncHI2-ST1 plasmid pCE1537-A with similar plasmid sequences retrieved from GenBank. In the key, ARG: antibiotic-resistance gene, VAG/MRG: virulence associated gene/metal resistance gene, MGE: mobile genetic element and HP: hypothetical protein.

**Figure 5:**
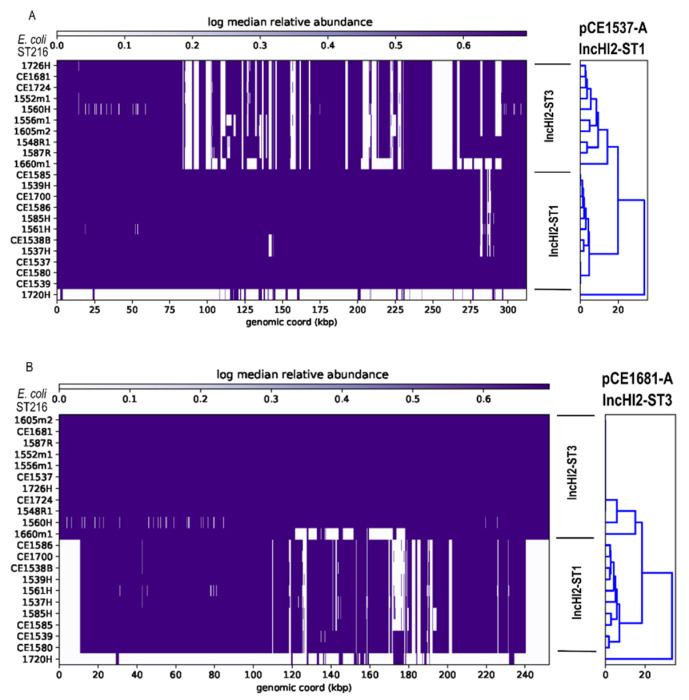
Heat maps showing the distribution of reference plasmids within sequenced short reads of *E. coli* ST216 isolates from silver gulls at Five Islands. Blue colour indicates the coverage profile of each short read with respect to the reference plasmid. Reference plasmids: (**A**) pCE1537-A (IncHI2-ST1) and (**B**) pCE1681-A (IncHI2-ST3).

In all *bla*_IMP-4_-positive *E. coli* ST216 isolates, *bla*_IMP-4_ was found on HI2-ST1 plasmids as a component of In809 that was flanked by IS*26* (Figure 3) (*n* = 11, Figure 4 and Figure 5A) or on HI2-ST3 plasmids flanked by IS*26* variant, IS*15DI* [62] (*n* = 10, Supplementary Figure S4-A and Figure 5B).

### 3.5. Plasmids Identified in ST216 Isolates

Among the 22 ST216 WGS, plasmid families HI2 (95%, *n* = 21) and FIB(K) (91%, *n* = 20) with F-:A13*:B-(*n* = 5), F-:A18*-:B- (*n* = 3), F100:A-:B- (*n* = 2) F2:A13*:B^-^ (*n* = 1), F2:A18*-:B- (*n* = 1) and F100:A13*-:B- (*n* = 1) were observed. Other plasmid replicons included FIA(HI1) (50%, *n* = 11), IncY (50%, *n* = 11), X5 (45%, *n* = 10), Col156 (*n* = 9, 41%), R (32%, *n* = 7), Col4401, FII and Q1 (18%, *n* = 4), X1 (14%, *n* = 3) and Col(BS512) (4.5%, *n* = 1) (Supplementary Table S1). Matches to Q1 plasmids need to be interpreted with caution as transposons such as Tn*6029* and Tn*6026* contain a Q1 *rep* and these transposons are encountered frequently [63]. Plasmid profiles ranged from one to eight plasmids among the 22 ST216 genomes. HI2 family plasmids were of two sequence types including HI2-ST1 (50%, *n* = 11) and HI2-ST3 (45%, *n* = 10) and all carried *bla*_IMP-4_ (Supplementary Table S1). Interestingly, the HI2-ST3 plasmid was observed with a single acquired N plasmid *rep* gene, giving an apparent hybrid plasmid type.

Phylogenetic analysis of closed plasmids revealed a higher diversity in the MDR region of HI2-ST1 plasmids compared to HI2-ST3 plasmids (Figure 5A,B, respectively). With the exception of 1560H showing variability in its plasmid backbone sequence and 1660m1 with a variable MDR region, HI2-ST3 plasmids were highly homologous (Figure 5B). Similarly, R plasmids were indistinguishable among the gull isolates except for isolate 1605m2 which showed sequence deviation in its plasmid backbone (Supplementary Figure S3D). Isolate 1605m2 had variable plasmid, ARGs and VAGs profiles and shared common *fimH* 69 and O154:H4 types with nine and six ST216 isolates, respectively. However, *sul3*, *cmlA1* and *dfrA12* were the only ARGs found in 1605m2 (Supplementary Table S1). FIB(K) and FIA plasmids were not typeable while three FII plasmids belong to ST2 (*n* = 2) and ST100 (*n* = 1) (Supplementary Figure S1). The MDR region in FIB(K) plasmids, observed in six isolates, showed high sequence identity (Supplementary Figure S3B). Plasmids Col156, which carry the colicin E7 operon (Figure 2) and X5 that carry flagellar transcriptional modulator gene (*flhD*) and haemolysin expression modulator (*hha*) (Supplementary Figure S4-C) were distributed among *E. coli* ST216 isolates with a prevalence of 41% (*n* = 9) and 45% (*n* = 10), respectively (Supplementary Figure S3C,E).

### 3.6. Analysis of HI-ST1, HI2-ST3, FIA, FIB(K), X5, R and Col156 Plasmids

Twelve complete and closed plasmids including HI2-ST1, HI2-ST3, FIA, FIB(K), X5, R, Y, Col156, ColE and Col440I were generated by long read sequencing of isolates CE1681 (*n* = 6) and CE1537 (*n* = 6) (Supplementary Table S2). From those 12 plasmids, only five (pCE153-A, pCE1681-A, pCE1681-B and pCE1681-D) carried antibiotic resistance genes while two (pCE1681-C and pCE1537-C) carried the colicin E7 operon (Supplementary Table S2).

HI2-ST1 plasmid pCE1537-A had a backbone structure typical of HI2 plasmids, an Inc type broadly identified previously in 350 commensal *E. coli* from Australian swine (Figure 4) and was shown to be conjugative after its conjugation into a recipient *E. coli* [64]. It carried a complex resistance locus that included genes for resistance to aminoglycosides (*aac(3)-IId*, *aph(3’’)-Ib*, *aac(6’)-Ib3*, *acc(6’)-Ib-cr* and *aph(6)-Id*), beta-lactams (*bla*_IMP-4_, *bla*_OXA-1*,*_ *bla*_TEM-1b_ and *bla*_SHV-12_), fluoroquinolones (*aac(6’)-Ib-cr*), rifampicin (*arr-3*), phenicol (*catA2* and *catB3*), sulphanomides (*sul1*), tetracycline (*tet*(D)) and macrolides (*mph*(A)) (Supplementary Table S2). A large portion of the resistance region forms a pseudo-compound transposon flanked by copies of IS*26* in the same orientation and included other IS elements (Figure 3). The carbapenemase gene *bla*_IMP_-_4_ was observed as a gene cassette in an In809 class 1 integron (Figure 3). Analysis of metal resistance operons identified genes associated with resistance to arsenic, mercury, tellurium, lead, copper, cobalt, zinc, cadmium as well as several multidrug efflux transporters (Figure 4). Moreover, plasmid pCE1537-A contained resistance genes for formaldehyde (*frmB* and *frmR*) and ethidium bromide (*emrE*) (Figure 4).

HI2-ST3 plasmid pCE1681-A (Supplementary Table S2 and Supplementary Figure S4-A) carried genes that confer resistance to aminoglycosides (*aac*(6’)-*Ib-cr, aac(3)-IId),* beta-lactams (*bla*_IMP-4_, *bla*_OXA-1_ and *bla*_TEM-1b_), fluoroquinolones (*aac*(6’)-*Ib-cr*), rifampicin (*arr-3*), macrolides (*mph*(A)), phenicol (*catB3*), sulphanomide (*sul1*) and tetracycline (*tet*(A)). Two class one integron structures were identified in pCE1681-A (Supplementary Figure S4-A). The first contained *mph*(A)*, sul1, catB3, bla*_OXA-1_ and *aac*(6’)-*Ib-cr* and several IS elements. The second, much smaller structure harboured *bla*_IMP-4_ hosted in the integron, flanked by inward-oriented IS*6* elements, with noted mutations for variant IS*15DI* [62]. MRGs for mercury and tellurium were also detected on pCE1681-A (Supplementary Figure S4-A).

FIA plasmid pCE1537-B found in isolate CE1537 did not harbour ARGs but carried a flagellin (*fliC*) encoding an Hx allele and a *fliC* repressor gene. These genes were flanked by an IS630-like element and ISSen4 (Supplementary Table S2 and Supplementary Figure S4-B). Y plasmid pCE1537-E accommodated an intact phage (Escher_RCS47_NC_042128) which was flanked by an IS*26*-like element upstream and an IS*26* element downstream of its sequence.

**Figure 6:**
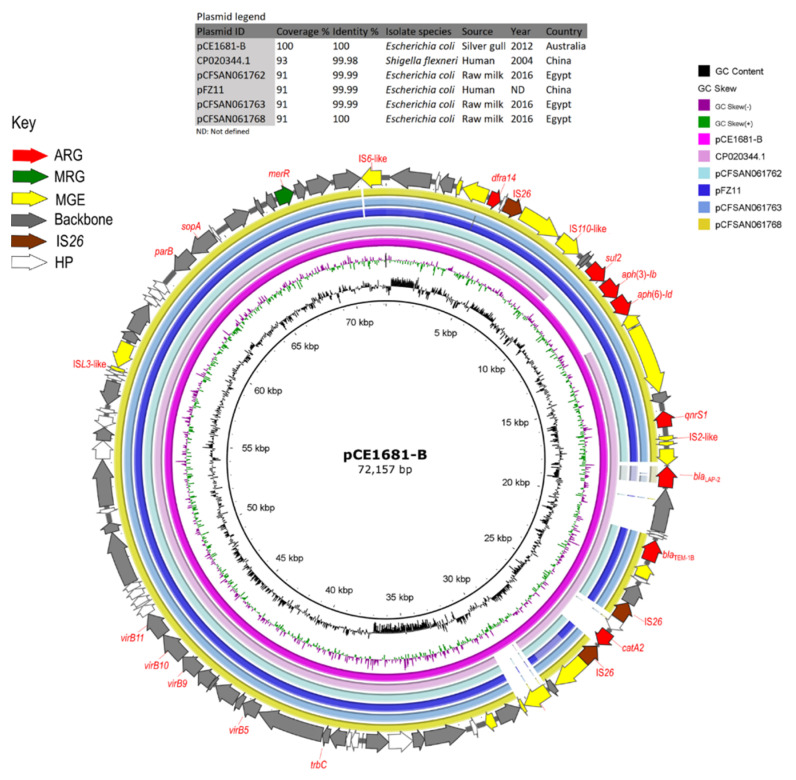
BRIG comparison of *qnrS1* positive IncFIB(K) plasmid pCE681-B with similar plasmid sequences retrieved from GenBank. In the key, ARG: antibiotic-resistance gene, MRG: metal resistance gene, MGE: mobile genetic element and HP: hypothetical protein.

FIB(K) plasmid pCE1681-B, found in isolate CE1681 harboured genes for resistance to aminoglycoside (*aph*(6)*-Id* and *aph*(3′′)*-Ib*), beta-lactam (*bla*_LAB-2_ and *bla*_TEM-1b_), fluoroquinolones (*qnrS1*), phenicol (*cat*A2), sulphanomide (*sul2*) and trimethoprim (*dfrA14*). All the ARGs were part of an atypical class 1 integron structure that included four IS*26* elements (Figure 6).

Only a single ARG (*bla*_TEM-1b_) and several arsenic MRGs were identified on IncR plasmid pCE1681-D (Supplementary Table S2 and Figure S4-D). X5 plasmid pCE1681-E harboured several formaldehyde detoxification genes as well as *flhD* and *hha* (Supplementary Figure S4-C). Col156 plasmid pCE1681-C contained a DNase-bacteriocin operon encoding colicin E7 (Figure 2) [65].

Based on preset criteria (Table 2) four plasmids were selected for comparison with pCE1537-A (HI2-ST1). HI2 plasmids, pAUSMDU8141-1, pC15_001, pIMP4-SEM1 and pMS7884A showed high sequence identity in their backbone sequences but variable MDR regions. They all originated from Australia and two of them (pIMP4-SEM1 and pMS7884A) carried *bla*_IMP-4_ (Figure 3 and Figure 4 and Table 2). However, these four plasmids (Table 2) were identified from different bacterial species from different geographic locations with pAUSMDU8141-1, pC15_001 and pMS7884A originating from clinical sources in Victoria, Sydney and Brisbane, respectively. pMS7884A is from *Enterobacter hormaechei* strain MS7884A that carried *bla*_IMP-4_ while pC15_001, also from *E. hormaechei*, harboured *bla*_OXA-1_. Both plasmids were associated with clinical outbreaks in the Intensive Care Unit (ICU) of Concord Repatriation Hospital in Sydney between 2006 and 2015 [66] and an ICU and burns facility at a Brisbane hospital in 2015 [67], respectively. Plasmid pIMP4-SEM1 originated from a *Salmonella enterica* isolate colonising a feline companion animal [68].

A comparison of the *bla*_IMP_-_4_-containing In809 integron and IS*26*-flanked regions within MDR genes of pCE1537-A, pMS7884A (clinical source), pIMP4-SEM1 (companion animal source) and pC15_001 (clinical source) revealed high sequence identity (Figure 3). Translocations of *qacG* and *qacE* were evident between pCE1537-A and pMS7884A plasmids and an IS*Vsa3* transposase (IS*91* family) replaced *bla*_IMP-4_ on pC15_001 (Figure 3). We did not perform comparative analyses with pCE1681-A (HI2-ST3 plasmid).

pCE1681-B shared high sequence coverage and identity with five plasmids (Table 2). Four of these pCFSAN061762, pFZ11, pCFSAN061763 and pCFSAN061768 were from *E. coli* and CP020344.1 was from *Shigella flexneri*. These five plasmids carried *qnrS1*, *bla*_TEM-1b_ and *dfrA14* (Figure 6). Plasmids CP020344.1 and pFZ11 had a clinical source in China while plasmids pCFSAN061762, pCFSAN061763 and pCFSAN061768 originated from raw milk in Egypt (Table 2).

Analysis of plasmid pCE1681-C showed 100% coverage and ≥ 99% identity with two *E. coli* plasmids (Table 2), all carrying a colicin E7 operon (Figure 2). Plasmids ColE7-K317 and pECAZ146_5 originated from an unidentified source in Pakistan and from a clinical source in Italy, respectively.

Plasmid pCE1681-E showed 86% coverage and 100% identity to a *bla*_IMP-4_-positive X5 plasmid pEc1677 (Supplementary Figure S4-C) originating from an *E. coli* isolate from a silver gull in Sydney [69]. The two plasmids had a similar backbone structure but differed in their variable region with the absence of ARGs in pCE1681-E while pEc1677 harboured *bla*_IMP-4_ and other ARGs. In plasmid pEc1677 *bla*_IMP-4_ was part of a class 1 integron that included antibiotic resistance genes *aac*(6′)-*Ib3*, *catB3* and *sul1* and quaternary ammonium resistance genes *qacG* and *qacE.* The organisation of these genes was like that observed in pCE1537-A (HI2-ST1) (Figure 4). Both plasmids, pEc1677 and pCE537-A shared an IS*26* element upstream of *bla*_IMP-4_ while they had IS elements IS*4321* and IS*91*-like bordering *sul*1. 

## 4. Discussion

Wild and urban-adapted birds carry, cycle, and transmit mobile elements carrying ARGs and VAGS between humans, animals and the environment [3,35,36,70,71]. Carriage of MDR bacteria by wild and urban bird populations remind us of the need to remove anthropogenic pollutants, particularly antibiotic resistant bacteria, antibiotic residues, disinfectants and metals from the environment.

*E. coli* with variable MDR profiles including carbapenem resistance encoded by *bla*_IMP-4_ carried on HI2-ST1 and HI2-ST3 plasmids have been recovered from cloacal samples of silver gulls in Australia [34]. Based on this report, we utilised WGS to detect and investigate *E. coli* that carry genes encoding resistance to CIA. After ST457 [35], *E. coli* ST216 represented the second most common *E. coli* ST accounting for 8.4% of all *E. coli* isolates (*n* = 262) obtained from cloacal samples at Big Island and 5% of the 448 *E. coli* recovered from the three coastal sampling sites (Five Islands, Sydney and Montague Island) in New South Wales, Australia. SNP-based phylogenetic analysis of international ST216 isolates (Figure 1) divided ST216 into two clades. Clade I is small and includes isolates of clinical and domestic animal origin in addition to isolates sequenced here from silver gulls (SCG1). ST216 genomes in SCG1 show a clonal-like distribution and many strains carry HI2-ST1 plasmids that are highly similar to HI2-ST1 plasmids observed in Australian clinical and companion animal sources (Figure 4). Documented variability in the carriage of genetic cargo residing in complex resistance regions of HI2-ST1 plasmids in SCG1 (Figure 5A) suggest that multiple plasmid acquisition events may occur or that the resistance regions respond rapidly to selection pressures where ST216 persists. 

The phylogeny of ST216 genomes in SCG2 (Figure 1) indicates that *E. coli* ST216 is globally distributed and occupies a diverse host range, including isolates from environmental sources as well as from humans, domestic animals and wildlife (Supplementary Figure S1). The distribution of SCG2 into several subphylogenetic groups (Figure 1) coupled with the diverse plasmid content compared with SCG1 (Figure 1 and Supplementary Table 1) suggest multiple introduction events of ST216 in silver gulls. 

The risk of dissemination of *E. coli* ST216 carrying *bla*_IMP-4_ and genes encoding resistance to other CIA to humans and domestic animals is a cause for concern. Silver gulls in the Sydney-Wollongong region share common spaces that include facilities with high human contact (rail and bus stations and municipal parks) and ST216 has been isolated from silver gulls in different geographic regions of Australia [72]. Plasmids carrying multidrug resistance genes with different incompatability markers have been recovered from multiple *E. coli* and *Salmonella enterica* lineages from gulls in Australia [34,35,36,72]. *E. coli* ST216 show a clear propensity to acquire plasmids that carry diverse resistance gene cargo and are linked to aquatic environments where they have been responsible for recalcitrant, carbapenem-resistant infections in hospital drainage waste systems [24]. ST216 belongs to commensal phylogroup A. Apart from the outbreak cluster in the UK, most reports of ST216 in humans are sporadic cases [25,30] in hospital. There is a clear association of *E. coli* ST216 with wildlife and reports to date all describe carriage of genes encoding resistance to CIA [33,72] [This study]. Given its comparatively low reporting in humans and its repeated links with wildlife and aquatic environments it is tempting to speculate that water may be a natural reservoir for *E. coli* ST216 and aquatic wildlife hosts, particularly birds, for its distribution. The pressing question is what role wildlife will play, if any, in the continued evolution of *E. coli* ST216 given the propensity for it to acquire self-replicating mobile genetic elements.

The ARG content in our ST216 population (Supplementary Table S1) is largely due to the carriage of HI2 (ST1 and ST3) and FIB(K) plasmids (Supplementary Table S2). Isolation of Enterobacterales from gulls using antibiotic selection likely created a bias in what lineages were observed and explains the high carriage of plasmids and ARGs we found in the silver gulls. Comparisons to strains not isolated under an antibiotic selective pressure will be necessary to reveal the distribution of the now identified AMR genes and their transfer mechanisms. Insertion sequences IS*26* and IS*15DI* play a major role in capturing and mobilising antibiotic resistance genes [62,73,74] and are often found in close association with class 1 integrons [64,75,76,77]. The presence of these insertion elements serves as a hotspot for capture of ARGs flanked by IS*26* [73]. IS*26* also plays an important role in altering the structure of class 1 integrons by truncating the 3’-CS and the 3´ end of *intI1* [77,78,79]. IS*26* has played a role in shaping the resistance regions in HI2 ST1 and ST3 and F plasmids that are carried by *E. coli* ST216 from silver gulls described in this study. IS*26* elements can facilitate hybrid plasmid formation [16,80] and may have a role in promoting plasmid stability [81]. Comparative analysis of HI2-ST1 plasmid pCE1537-A provides evidence that plasmids circulate closely within different bacterial species and hosts (humans, companion animals and wildlife) in Australia. It is also concerning that IS*26* has been implicated in the mobilisation of virulence genes [82].

F plasmids carrying ARGs and VAGs are widespread and are commonly associated with Enterobacterales from clinical sources and food-animals [83,84]. Isolates from silver gulls carry various VAGs, metal transport systems and the colicin E7 operon on several plasmids. The acquisition of these genetic elements can enhance the survival, colonisation and persistence characteristics of bacteria that inhabit different niches [65,85,86,87,88]. Moreover, the presence of both metal resistance genes and ARGs leads to coselection and promotion of ARGs in bacterial populations in the absence of antibiotics [89]. These data suggest that the evolution of ST216 and their mobilome are influenced by anthropogenic pollution. Feeding and flight behaviours [90] are likely to have a profound influence on the silver gull resistome.

At the time of writing, a total of 137 ST216 isolates were deposited in the Enterobase database. Of these 121 (80%) were deposited after 2010 with a global distribution across clinical, environmental, and animal hosts. Only a few of the ST216 isolates were collected from clinical samples associated with diarrhoea (ESC_FB7867AA and ESC_FB7867AA) in China and septicaemia (ESC_AA2218AA) in Germany. The increased frequency of reports of *E. coli* ST216 is concerning, particularly in light of their ability to acquire multiple diverse plasmids carrying ARGs, VAGS, biocins and metal resistance genes and their ability to colonise the gastrointestinal tracts of wild and urban birds. These characteristics are known to be important in the evolution of successful MDR bacterial clades [91]. However, unlike many dominant bacterial clades that show high genetic conservation within a geographical region [14], ST216 isolates appear to be phylogenetically interspersed and distanced from each other even within a single geographical location, suggesting that flight behaviour is an important attribute in understanding how wildlife, particularly birds, acquire drug-resistant flora. Based on these observations *E. coli* ST216 warrants further monitoring in bird populations in Australia and internationally. Furthermore, studies of the enterobacterial populations in urban-adapted and wild bird species using nonselective approaches is needed to improve understanding of lineages that colonise and persist in the avian gut. We also advise adopting a one health approach and investigating ST216 populations in silver gulls, humans and their surrounding environment to understand the transmission pathways and other features that influence pathogen evolution.

## 5. Conclusions

ST216 is a broad host range phylogroup A *E. coli.* Here we report the carriage of MDR *E. coli* ST216 by silver gulls on Five Islands near Wollongong, Australia harbouring diverse plasmids that carry multiple ARGs, VAGs and metal resistance genes. Most of the ST216 isolates were carbapenemase producers and carried *bla*_IMP-4_ on HI2-ST1 and HI2-ST3 plasmids. ARGs within HI2 plasmids were assembled in complex resistance regions together with metal resistance genes and multiple copies of IS elements including IS*26* and IS*26* derivative IS*15DI*. We report the spread of highly related IncHI2-ST1 plasmids between various bacterial hosts from different sources that include humans, domestic animals and wildlife in Australia. The recent increase of global ST216 reports isolated from different sources, expressing ARGs for critically important antibiotics and causing long lasting clinical outbreaks are concerning. To understand the transmission cycle of ARGs and MDR bacteria and its associated human risk, it is essential to adopt a one health approach that take into consideration all aspects of the ecological system with a focus on intermediate hosts (as gulls) that can act as vectors and sentinels for the spread of ARGs. Another consideration is the importance of interactions between different bacterial species mediated by mobile genetic elements such as HI2 plasmids and IS*26* and its effect on the evolution and pathogenicity of these organisms.

## Figures and Tables

**Table 1:** *Escherichia coli* ST216 short read and long read sequences, metadata and quality control statistics.

Sequence ID	Sequence Type	Enterobase Barcode No.	GenBank Accession No.	Coverage	Total Sequences	Poor Quality Sequences	Sequence Length (bp)	GC%
1556m1	SR	ESC_QA8784AA	JAEUYL000000000	95×	1,226,163	0	151	51
CE1586	SR	ESC_QA8782AA	JAEUYK000000000	72×	1,588,914	0	151	51
1548R1	SR	ESC_QA8786AA	JAEUXR000000000	79×	1,386,478	0	151	51
1552m1	SR	ESC_QA8785AA	JAEUXS000000000	83×	1,425,868	0	151	51
CE1585	SR	ESC_RA0975AA	JAEUXT000000000	143×	2,366,204	0	151	51
1587R	SR	ESC_RA0998AA	JAEUXU000000000	84×	1,440,780	0	151	50
CE1724	SR	ESC_RA0997AA	JAEUXV000000000	76×	1,286,453	0	151	51
CE1681	SR	ESC_RA0995AA	JAEUXW000000000	73×	1,367,751	0	151	51
1605m2	SR	ESC_RA0996AA	JAEUXX000000000	81×	1,354,070	0	151	51
1660m1	SR	ESC_QA8910A	JAEUXY000000000	77×	1,298,542	0	151	51
CE1700	SR	ESC_QA8911AA	JAEUXZ000000000	74×	1,254,843	0	151	51
1537H	SR	ESC_QA8912AA	JAEUYA000000000	127×	2,206,779	0	151	52
1720H	SR	ESC_QA8787AA	JAEUYJ000000000	16×	221,227	0	151	51
1726H	SR	ESC_QA8788AA	JAEUYI000000000	58×	974,309	0	151	51
1560H	SR	ESC_QA8790AA	JAEUYH000000000	61×	1,042,416	0	151	51
1561H	SR	ESC_QA8789AA	JAEUYG000000000	43×	729,472	0	151	51
1539H	SR	ESC_QA8791AA	JAEUYF000000000	106×	1,912,588	0	151	51
1585H	SR	ESC_QA8795AA	JAEUYE000000000	499×	9,553,397	0	151	51
CE1539	SR	ESC_QA8792AA	JAEUYD000000000	161×	2,911,482	0	151	51
CE1580	SR	ESC_QA8794AA	JAEUYC000000000	358×	7,231,680	0	151	52
CE1538B	SR	ESC_QA8793AA	JAEUYB000000000	123×	2,115,704	0	151	51
CE1537	GLR	ND	JABBCF000000000	203×	462,216	0	51-197802	50
CE1537	PLR	ND	ND	317×	98,607	0	50-103991	46

SR: short read, GLR: genome long read, PLR: plasmids long read and ND: not defined.

**Table 2:** Characteristics of plasmids used for comparison with plasmids pCE1537-A, pCE1681-B, pCE1681-E, and pCE1681-C.

Plasmid ID	Comparison Plasmid	GenBank Accession no.	Coverage (%)	Identity (%)	Replicon	Species	Source	Country	Region	Year
pCE1537-A	pAUSMDU8141-1	CP022696.1	100	99.77	IncHI2-ST1	*Citrobacter farmeri*	Human	Australia	Victoria	2015
	pC15_001	CP042489.1	99	99.31	IncHI2-ST1	*Enterobacter hormaechei*	Human	Australia	Sydney	2009
	pIMP4-SEM1	KX810825.1	96	99.73	IncHI2-ST1	*Salmonella enterica*	Cat	Australia	ND	ND
	pMS7884A	CP022533.1	91	99.71	IncHI2-ST1	*Enterobacter hormaechei*	Human	Australia	Brisbane	2015
pCE1681-B	CP020344.1	CP020344.1	93	99.98	IncFIB(K)	*Shigella flexneri*	Human	China	Hangzhou	2004
	pCFSAN061762	CP042902.1	91	99.99	IncFIB(K)	*Escherichia coli*	Raw milk	Egypt	ND	2016
	pFZ11	KY051550.1	91	99.99	IncFIB(K)	*Escherichia coli*	Human	China	Fujian	ND
	pCFSAN061763	CP042900.1	91	99.99	IncFIB(K)	*Escherichia coli*	Raw milk	Egypt	ND	2016
	pCFSAN061768	CP042974.1	91	100	IncFIB(K)	*Escherichia coli*	Raw milk	Egypt	ND	2016
pCE1681-C	pCE1537-C	MT162141.1	100	99.83	Col156	*Escherichia coli*	Silver gull	Australia	Sydney	2012
	ColE7-K317	KJ470776.1	100	99.54	Col156	*Escherichia coli*	ND	Pakistan	ND	ND
	pECAZ146_5	CP018986.1	100	99.46	Col156	*Escherichia coli*	Human	Italy	Pisa	2012
pCE1681-E	pCE1537-C	MT162141.1	100	99.83	Col156	*Escherichia coli*	Silver gull	Australia	Sydney	2012
	pEc1677	MG516910.1	86	100	IncX5	*Escherichia coli*	Silver gull	Australia	Sydney	2012

ND: Not defined.

## Data Availability

Data presented in this study is available upon request. Part of this work was presented at the European Joint Programme (EJP) One Health conference in 2020 (talk number O:145).

## Supplementary Materials

The following are available online at https://www.mdpi.com/2076-2607/9/3/567/s1, Figure S1: SNP-tree showing clonal relationship and genetic characteristics of *Escherichia coli* ST216 isolates from silver gulls at five islands and international related isolates. Figure S2: SNP-tree showing clonal relationship and genetic characteristics of *Escherichia coli* ST216 isolates from silver gulls at Five Islands. Figure S3: Heat maps showing the distribution of reference plasmids within sequenced short reads of *Escherichia coli* ST216 isolates from silver gulls at Five Islands. Figure S4-A: Schematic diagram of plasmid pCE1681-A (IncHI2-ST3). Figure S4-B: Schematic diagram of pCE1537-B (IncFIA) plasmid. Figure S4-C: BRIG comparison of IncX5 plasmid pCE681-E with similar plasmid sequence retrieved from GenBank. Figure S4-D: Schematic diagram of pCE1681-D (IncR) plasmid. Table S1: Characteristics of sequenced *Escherichia coli* ST216 isolates from silver gulls at Five Islands, Table S2: Characteristics of sequenced closed and complete plasmids in *Escherichia coli* ST216 isolates CE1537 and CE1681 from silver gulls at Five Islands.
