# Supplementary material for: Epidemic HI2 Plasmids Mobilising the Carbapenemase Gene blaIMP-4 in Australian Clinical Samples Identified in Multiple Sublineages of Escherichia coli ST216 Colonising Silver Gulls
Source: Microorganisms. 2021 Mar 10;9(3):567. doi: 10.3390/microorganisms9030567 (PMC7999438; doi:10.3390/microorganisms9030567)
Supplement: Supplementary file 1 [file microorganisms-09-00567-s001.zip › microorganisms-1112325-sup/supplementary figures titles.docx]

**Supplementary Figures and tables:**

Table S1: Characteristics of sequenced *Escherichia coli* ST216 isolates from silver gulls at Five Islands. Boxes highlighted in yellow and + sign indicate the presence of gene or plasmid while empty boxes indicate the absence of gene or plasmid. In antibiotic susceptibility testing, boxes include the inhibition diameter (mm.) of tested antibiotic by disk diffusion method (AMP: ampicillin, S: streptomycin, S3: sulfonamides, TE: tetracycline, SXT: trimethoprim/sulfamethoxazole, C: chloramphenicol, KF: cefalotin, NA: nalidixic acid, CAZ: ceftazidime, CN: gentamicin, AMC: amoxicillin/clavulanic acid, CIP: ciprofloxacin, ETP: etrapenem, IPM: imipenem and MEM: meropenem). Colors of boxes are used as follows: complete resistance, orange; intermediate resistance, green; susceptibility, white. In MAST disk test, SC: suspected carbapenemase production and NR: no AmpC, ESBL or carbapenemase production. In carbapenemase production, boxes highlighted in green color and + indicate carbapenemase production. In IncFIB(K) ST: * indicate the nearest sequence type reported.

Table S2: Characteristics of sequenced closed and complete plasmids in *Escherichia coli* ST216 isolates CE1537 and CE1681 from silver gulls at Five Islands. Boxes highlighted in yellow and + sign indicate the presence of gene or plasmid while empty boxes indicate the absence of corresponding antibiotic resistance or other genes.


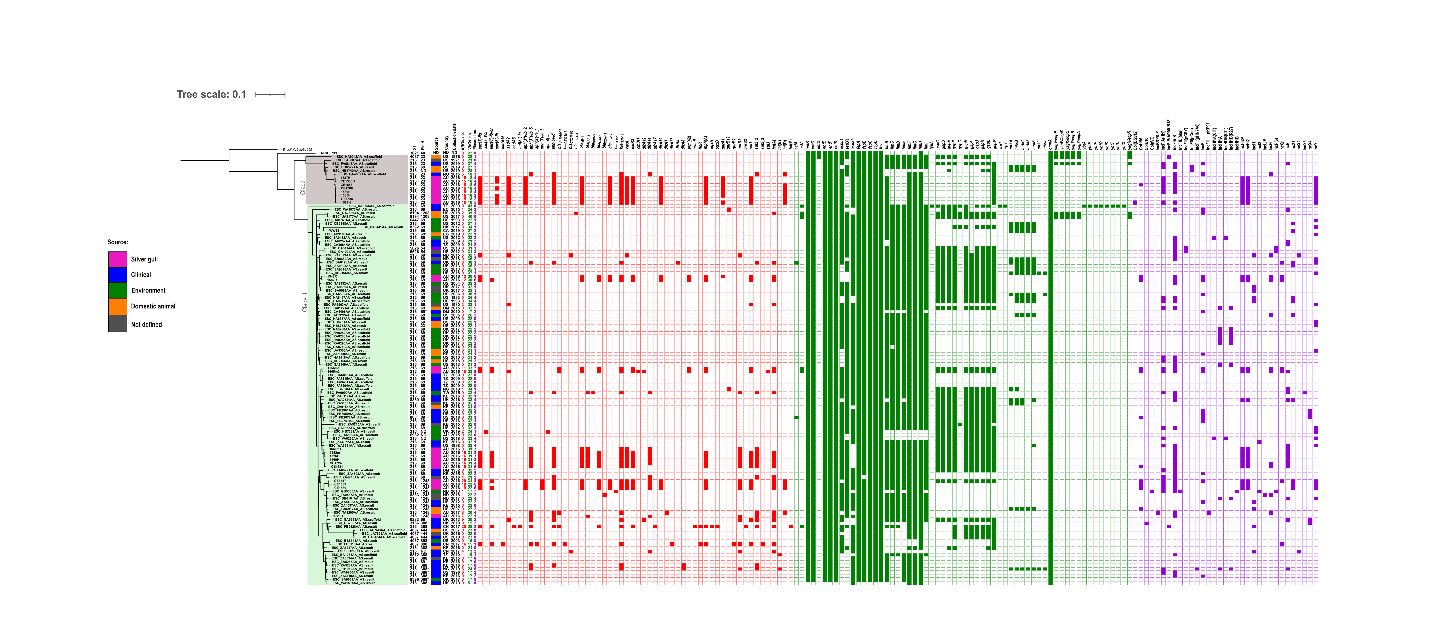


Figure S1: SNP-tree showing clonal relationship and genetic characteristics of *Escherichia coli* ST216 isolates from silver gulls at five islands and international related isolates. ARGs, VAGs and plasmids are indicated in red, green and violet colors, respectively. Presence of gene/plasmid is indicated by highlighting corresponding box.


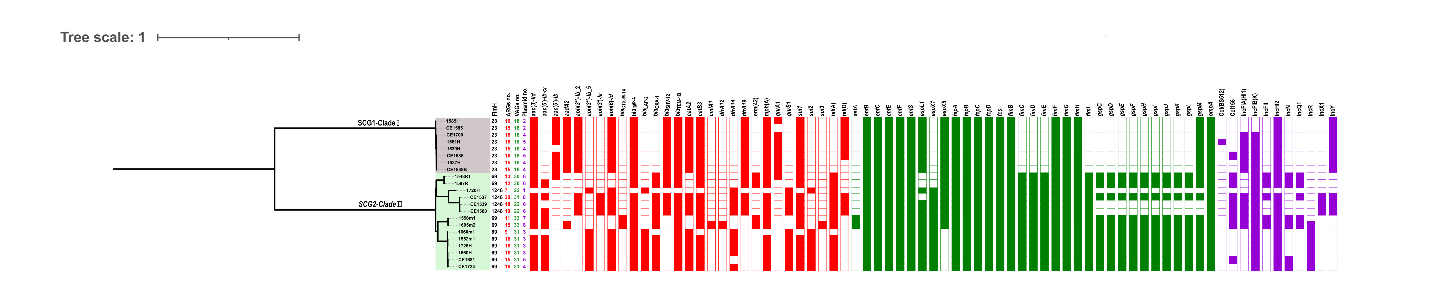


Figure S2: SNP-tree showing clonal relationship and genetic characteristics of *Escherichia coli* ST216 isolates from silver gulls at Five Islands. ARGs, VAGs and plasmids are indicated in red, green and violet colors, respectively. SCG1: SNP cluster group 1, SCG2: SNP cluster group 2.

S3-A


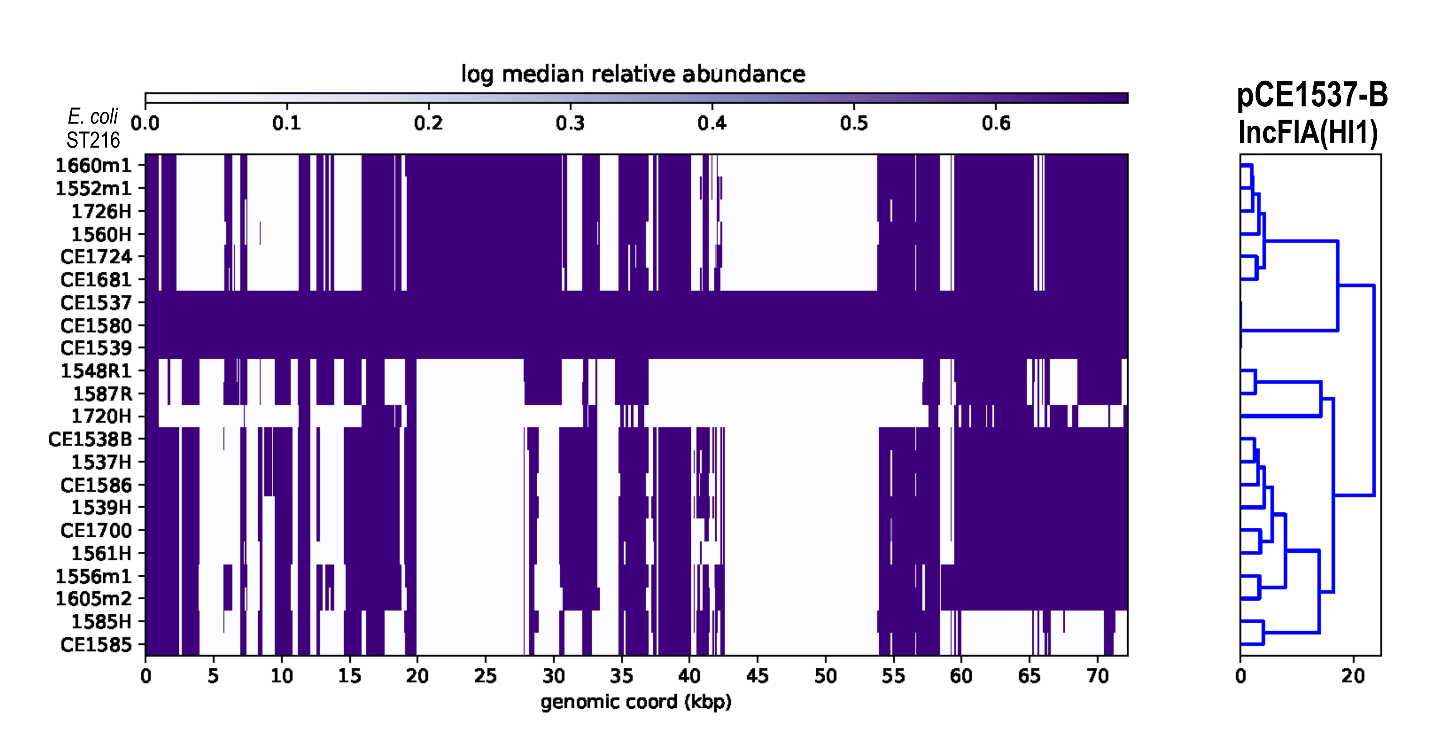


S3-B


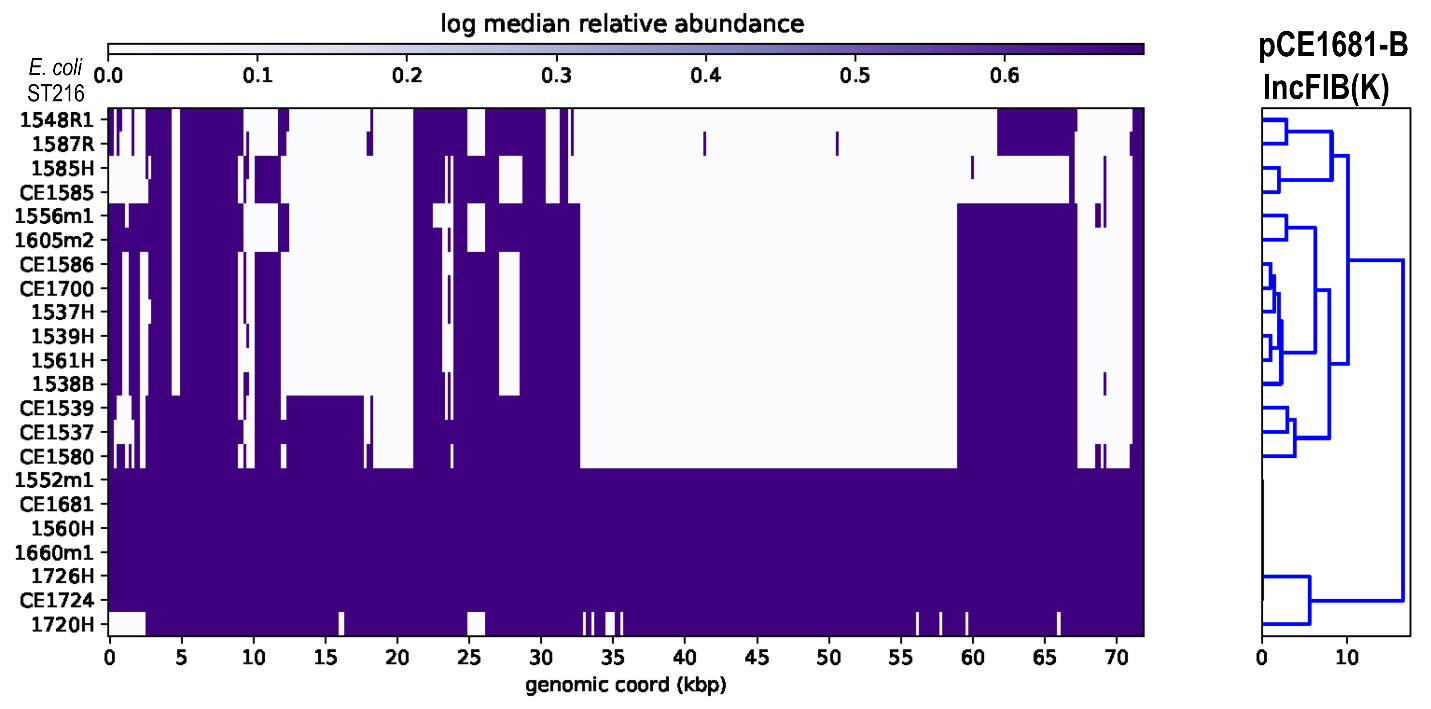


S3-C


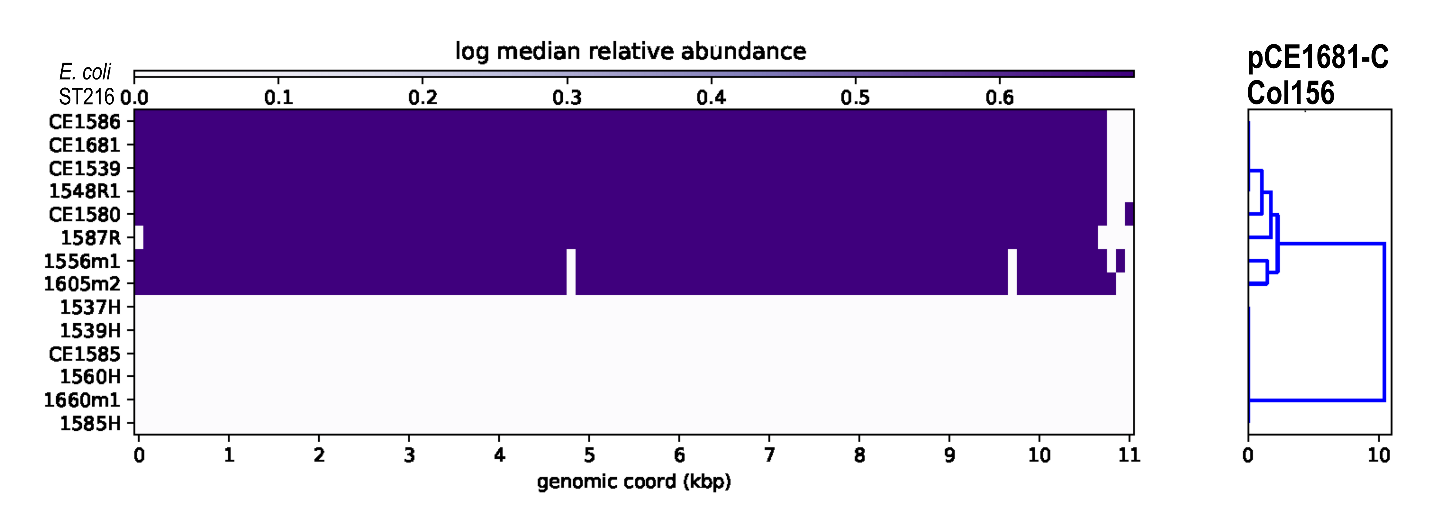


S3-D


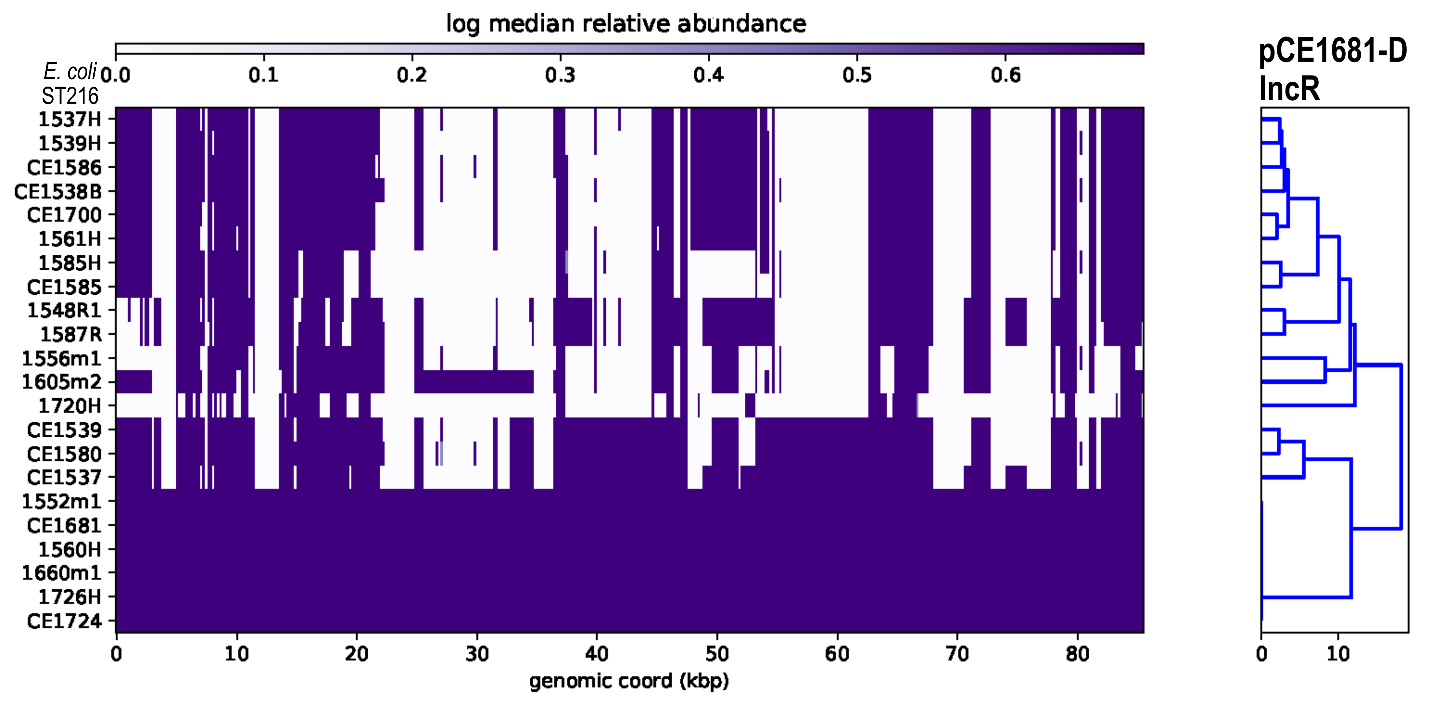


S3-E


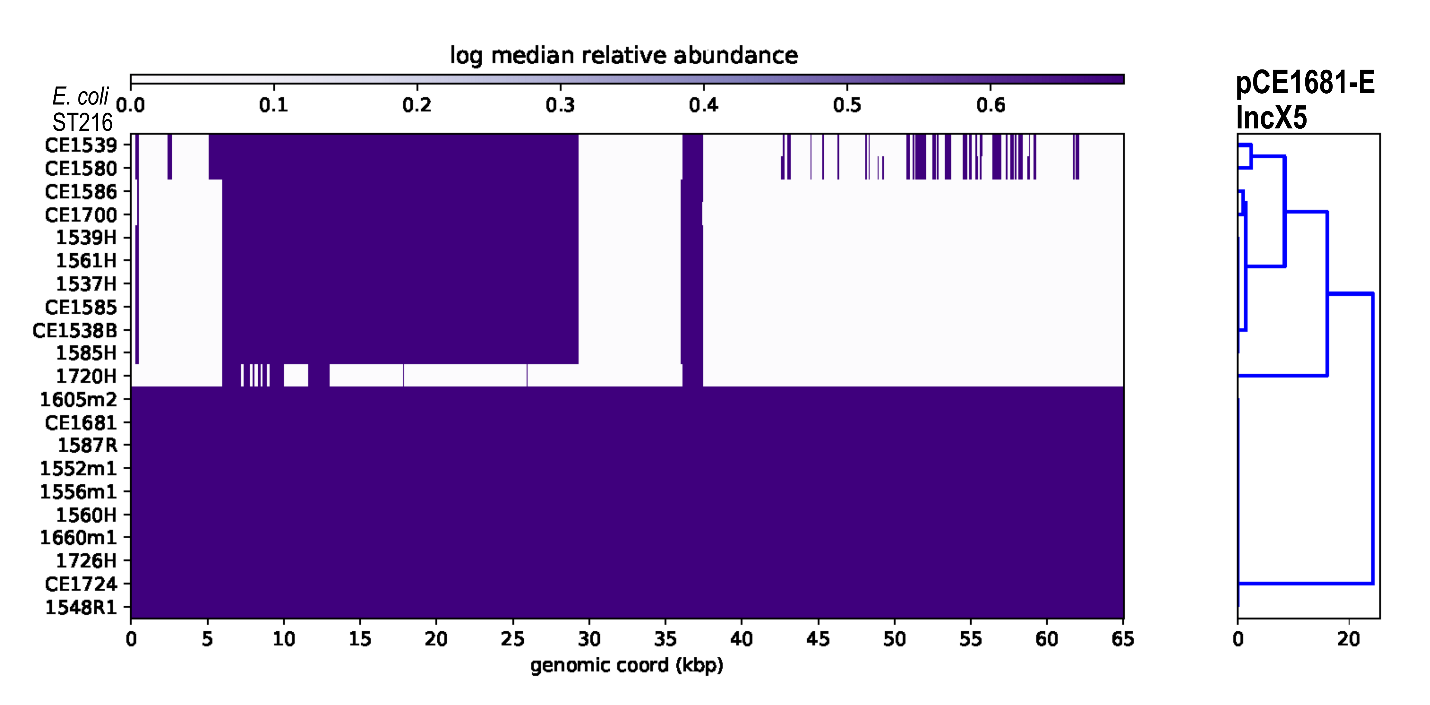


Figure S3 A-E: Heat maps showing the distribution of reference plasmids within sequenced short reads of *Escherichia coli* ST216 isolates from silver gulls at Five Islands. Blue color indicates the coverage profile of each short read with respect to the reference plasmid. Reference plasmids: S5-A: pCE1537-B (IncFIA(HI1)), S5-B: pCE1681-B (IncFIB(K)), S5-C: pCE1681-C (Col156), S5-D: pCE1681-D (IncR), S5-E: pCE1681-E (IncX5).


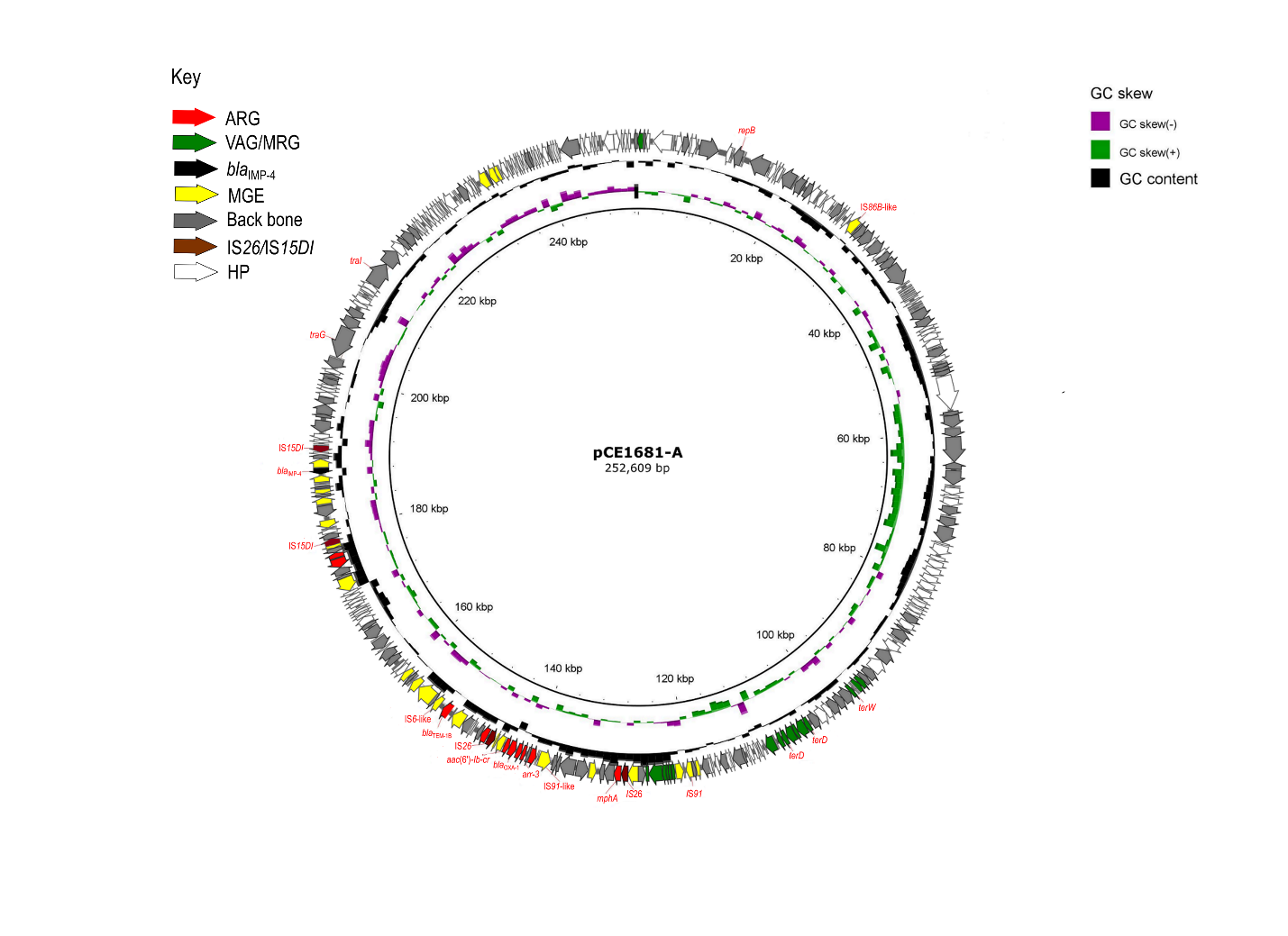


Figure S4-A: Schematic diagram of plasmid pCE1681-A (IncHI2-ST3). In the key, ARG: antibiotic-resistance gene, VAG/MRG: virulence associated gene/metal resistance gene, MGE: mobile genetic element and HP: hypothetical protein.


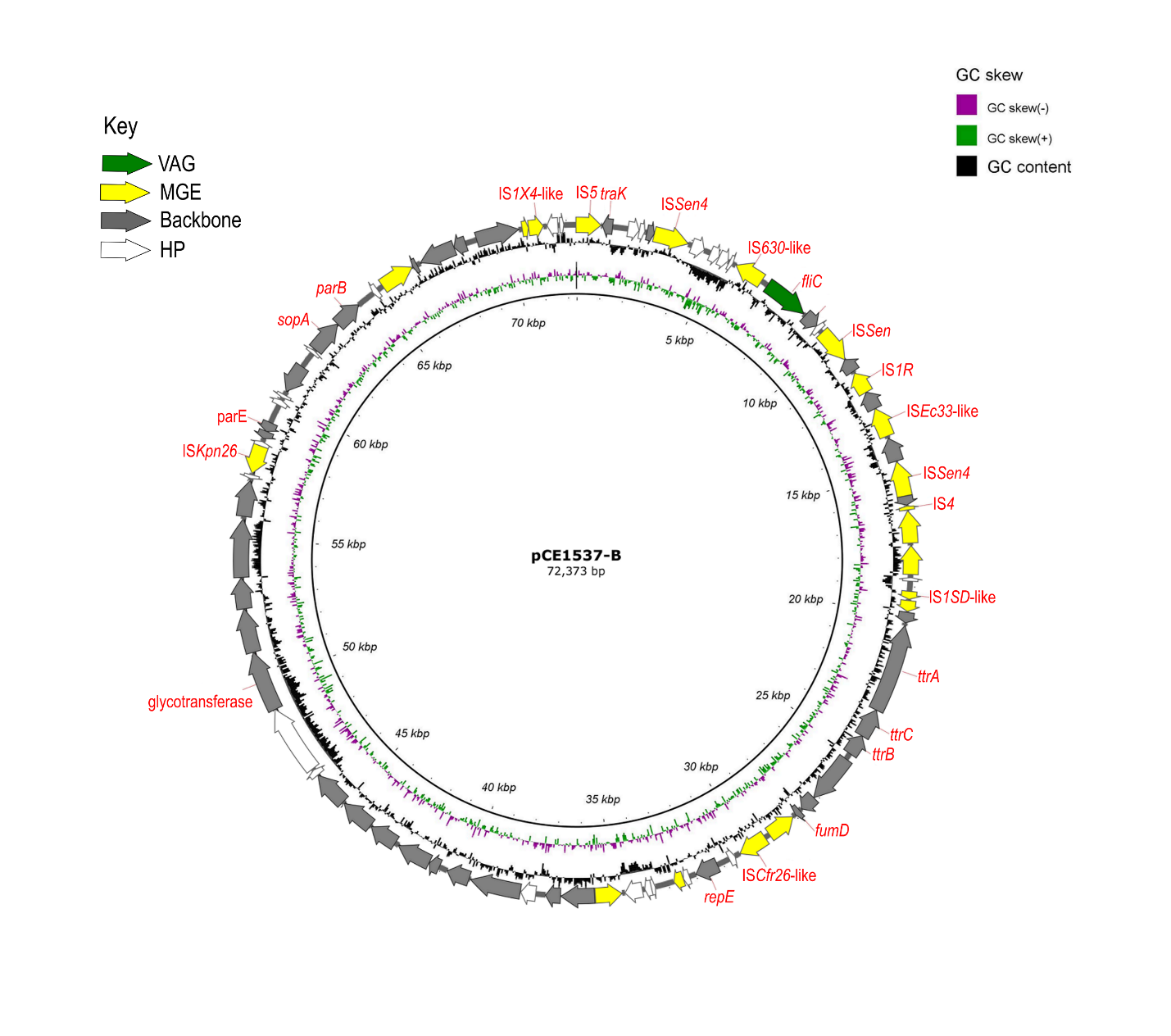


Figure S4-B: Schematic diagram of pCE1537-B (IncFIA) plasmid. In the key, VAG: virulence associated gene, MGE: mobile genetic element and HP: hypothetical protein.


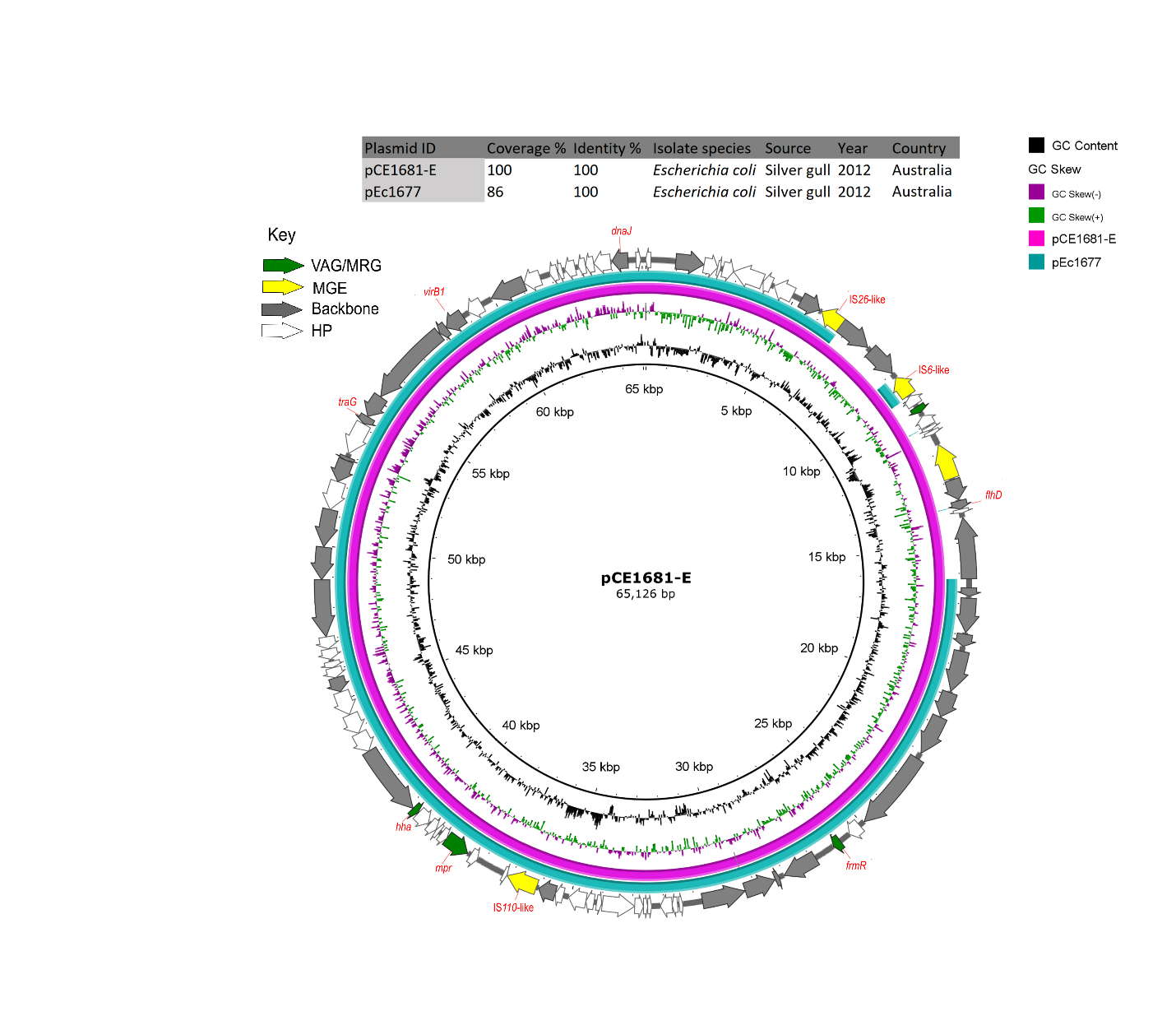


Figure S4-C: BRIG comparison of IncX5 plasmid pCE681-E with similar plasmid sequence retrieved from GenBank. *bla*_IMP-4_ positive plasmid pEc1677 originated from same colony of silver gulls at Five Islands in Australia were pCE1681-E was obtained. In the key, VAG/MRG: virulence associated gene/metal resistance gene, MGE: mobile genetic element and HP: hypothetical protein.


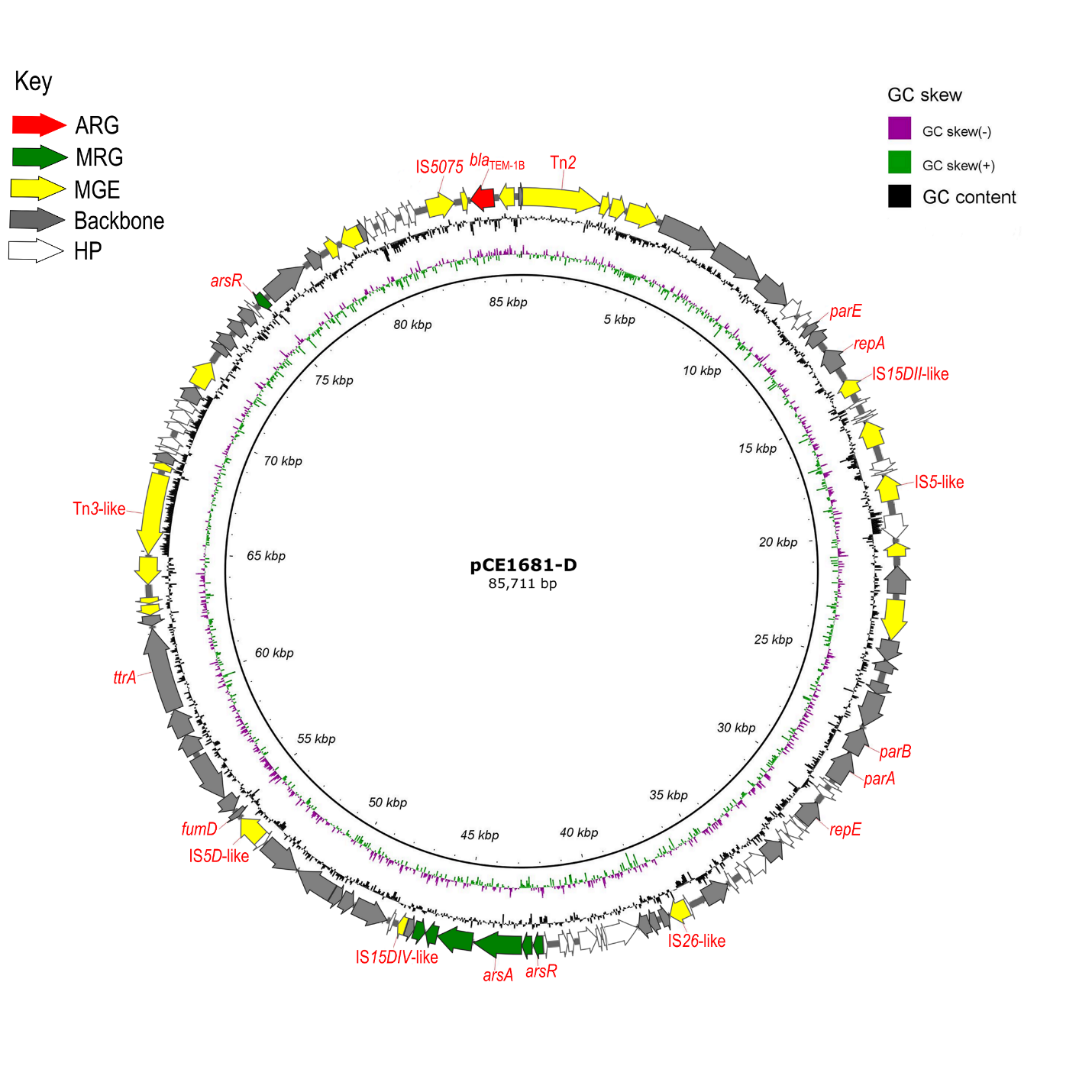


Figure S4-D: Schematic diagram of pCE1681-D (IncR) plasmid. In the key, ARG: antibiotic-resistance gene, MRG: metal resistance gene, MGE: mobile genetic element and HP: hypothetical protein.
